# Histological Analysis, Bioinformatics Profile, and Expression of Methylenetetrahydrofolate Reductase (MTHFR) in Bovine Testes

**DOI:** 10.3390/ani10101731

**Published:** 2020-09-23

**Authors:** Seth Yaw Afedo, Yan Cui, Sijiu Yu, Bo Liao, Zihan Zhao, Hui Li, Huizhu Zhang, Shengnan Zou, De Hong Li, Peng Zhang

**Affiliations:** 1 College of Veterinary Medicine, Gansu Agricultural University, Lanzhou 730070, China; afedosethyaw@gmail.com (S.Y.A.); sjyu@163.com (S.Y.); liaobo199303@163.com (B.L.); zzh406317387@163.com (Z.Z.); lihui198801@126.com (H.L.); alanbel0831@163.com (H.Z.); 18394171797@163.com (S.Z.); daboliaopang@163.com (D.H.L.); zhangpeng1010@163.com (P.Z.); 2 Gansu Province Livestock Embryo Engineering Research Center, College of Veterinary Medicine, Gansu Agricultural University, Lanzhou 730070, China

**Keywords:** MTHFR, bovine, bioinformatics, gene expression, testes

## Abstract

**Simple Summary:**

To date, several genes have been sequenced but their corresponding protein characteristics remain unknown. This study highlights the histological structure of bovine (yellow-cattle and yak) testes as a build-up to exploring the bioinformatics profile and expression characteristics of methylenetetrahydrofolate reductase (MTHFR) in bovine testes. Our findings suggest that yellow-cattle testis have similar anatomical characteristics with that of yak, except for the weight or size, for which that of yellow-cattle is significantly higher or greater than yak. We also found that the secondary and 3D protein structures of MTHFR were similar to that of humans, with differences in the number of nucleotides, amino acids, and some physico-chemical characteristics. Moreover, MTHFR mRNA expression was higher in adult yellow-cattle and yak compared to their juvenile ones, however, its protein expression was higher but not statistically significant in adult yellow-cattle and yak compared to the juvenile ones. This provides a basis for further investigations into the regulatory function of MTHFR in bovine testes.

**Abstract:**

Methylenetetrahydrofolate reductase (MTHFR), an enzyme expressed in mammalian testes, exerts a direct effect on spermatogenesis; however, its protein characteristics in bovine testes remain unknown. Here, we analysed bovine testicular structure, MTHFR bioinformatics profile, mRNA, and protein expression characteristics in yellow-cattle (y-c) and yak testis using histological procedures, bioinformatics analysis, qRT-PCR, and western blot. Testes from 13 bovines, ≤2 years juvenile (y-c, *n* = 3; yak, *n* = 3) and ≥3 years adult (y-c, *n* = 3; yak, *n* = 4) were collected and analysed. Anatomical characteristics of testis in y-c and yak were similar except the weight or size for which that of y-c was significantly higher or greater than yak. In y-c, an open reading frame (ORF) for 2600 nucleotides sequence, encoding 655 amino acids showed high homology with zebu cattle (99.51%) and wild yak (98.68%). Secondary and 3D protein structures were similar to that of humans with differences in the number of nucleotides, amino acids, and some physico-chemical characteristics. MTHFR mRNA expression in y-c and yak were significantly higher in adult testes compared with juvenile ones. However, its protein expression was higher, but not statistically significant, in adult y-c and yak compared to the juvenile ones. The highlights and inferences of these and other findings are discussed.

## 1. Introduction

Bovines are important livestock resources for agro-pastoralists around the world. They are used to produce meat, milk, and as work animals, thus constituting one of the most significant and indispensable animal resources. Domestic yellow-cattle (y-c, *Bos taurus*) and yaks (*Bos grunniens*) are multifunctional and dominant bovine livestock. In Siquan, Qinghai, Tibet, and Gansu provinces of China, these bovines are adaptable to extreme environmental conditions such as high altitudes above 3500 m, low oxygen content, low temperature, strong ultraviolet (UV) rays, the short period of grass growth, extreme cold, and other adverse conditions. Domestic y-c and yaks are usually cross-bred in most hybridization (breeding) programs to produce cattle-yak, a hybrid with the desired heterosis, although their males (F_1_ generation) are sterile [1,2,3,4,5]. For perpetuity of livestock species, maintenance of improved breeds, and for economic reasons, the fertility of male animals is of great concern to livestock owners, and over the years, several research studies have been conducted into detailed aspects of male fertility problems, including those relating to testicular morphology and genetics of spermatogenesis [6,7,8].

The genetics of spermatogenesis is highly complex, as phenotypes of testis histology are enormously heterogeneous. At least 2000 genes are involved in spermatogenesis [6]. Mammalian spermatogenesis is described as a continuum of cellular differentiation, comprising three fundamental phases: spermatogonia renewal/proliferation, meiosis, and spermiogenesis [9,10,11]. Spermatogenesis takes place in the seminiferous tubules of the testis, in close association with Sertoli cells (Scs), the somatic cells of the seminiferous epithelium [12,13]. Scs serve as sites for follicle-stimulating hormone (FSH) secretion by the pituitary gland through G-protein coupled receptors. FSH and testosterone are the most significant hormones controlling spermatogenesis. The proliferation of Scs is controlled by FSH during the perinatal and/or pubertal period, consequently, serving as a major determinant of adult spermatogenic capacity. Testosterone, the main androgenic steroid, is synthesized by Leydig cells (Lc) intratesticularly, and its synthesis is regulated by luteinizing hormones (LHs) [14,15]. In 2012, Šerý et al. reported that vesicle formation and/or exocytosis of LH might be interfered by C677T polymorphism of the MTHFR gene through the regulation of homocysteine metabolism [16]. C677T polymorphism could also influence, through its impact on the LH levels, the pathogenesis of different diseases like cardiovascular abnormalities [16,17].

MTHFR, an important regulatory enzyme for folate metabolism is well conserved and functionally preserved. In humans and mice studies, mutations and polymorphisms in MTHFR are associated with impaired spermatogenesis and male infertility. Three frequent polymorphisms (C677T, A1298C, and G1793A) of this gene are reported to cause reduced activity of MTHFR [16]. MTHFR is known to play a central role in DNA methylation and biosynthesis [18,19,20]. It converts folate into 5-methyltetrahydrofolate, a chief circulating form of folate in blood. MTHFR is also involved in the conversion of 5,10-methylenetetrahydrofolate to 5-methyltetrahydrofolate, a methyl donor for remethylation of homocysteine to methionine. Methionine, an essential amino acid, is then converted to S-adenosylmethionine, a methyl donor used in many reactions where substrates such as DNA, RNA, hormones, and lipids are methylated [21,22,23,24]. It has been shown that MTHFR deficiency, in addition to folate deficiency, hinders the methylation of a wide variety of substrates, including proteins, DNA, RNA, and histones because of decreased methionine supply [23,25]. Mutations and some polymorphisms are known causes of low MTHFR enzymatic activity. In male mice, inactivation of MTHFR results in hyperhomocysteinemia and infertility with abnormal testicular histology characterized by absence of germinal cells and spermatogenesis arrest [26]. Spermatogenesis may be particularly vulnerable to changes in the methyl pool brought about by deficiency in MTHFR. High levels of MTHFR expression in mammalian testes has been reported and in humans; its protein expression is high in the cells of the seminiferous tubules and Leydig cells [20]. It has also been reported that hypermethylation of promoter regions of MTHFR leads to nonobstructive azoospermia and potential idiopathic infertility in humans [27,28]. Furthermore, in human and mouse studies, a large body of controversial data exists regarding the epidemiological impact of frequent MTHFR variants as risk factors for conditions such as neural tube defect [29,30]. Reports on endogenous factors influencing bovine development and growth are rare, and no study has investigated MTHFR gene expression or protein characteristics in bovine testes and its exact functions [31]. Therefore, this study was conducted to analyse testicular histopathology, evaluate the full MTHFR coding sequence, amino acid sequence, mRNA and protein expression, and its predicted protein characteristics in bovine testes. Considering the significant role MTHFR play in DNA methylation, biosynthesis, and cell proliferation in other species, it was null hypothesized that the regulation and expression of MTHFR is the same in the testes of juvenile and adult y-c or yaks, and that changes in expression could impact testicular function or spermatogenesis. A more comprehensive understanding of MTHFR actions during biosynthesis, transcription, translation, and post-translational regulation during spermatogenesis in bovine testes will undoubtedly contribute to knowledge on the basic physiology of mammalian testes and spermatogenesis.

## 2. Materials and Methods

### 2.1. Reagents, Chemicals, and Instruments

Primers for MTHFR gene were synthesized by Beijing Genomics Institute (Beijing, China); Trizol reagent was purchased from Solarbio (Beijing, China); Go Script^TM^ Reverse Transcription (RT) System from Promega (Madison, WI, USA); DNA Ladder DL2000 was purchased from Gstar, (Chendu, China); rabbit anti-MTHFR antibody from Bioss (Beijing, China). LightCycler96 thermocycler was purchased from Roche (Mannheim, Germany), and SYBR Green Premix DimerEraser from Promega (Mannheim, Germany).

### 2.2. Animals and Tissue Sample Collection

Testicular samples used in this study were obtained from ≤2 years juvenile (y-c, *n* = 3, yak, *n* = 3) and ≥3 years adult (y-c, *n* = 3, yak, *n* = 4) from Wuwei and Linxia towns in Gansu province of China. Prior to slaughter, the animals grazed on natural pasture without feed supplementation and were declared healthy. All procedures and protocols involving tissues from animals were in accordance with the Animal Ethics Procedure and Guidelines of the People’s Republic of China as well as the Guide for the Care and Use of Experimental Animals. Procedures involving animal tissues were also approved by the Institutional Animal Care and Use Committee (IACUC) of Gansu Agricultural University with Permit No. GSAUAEC-2016-007.

Testes were collected from a local abattoir within 30 min of slaughter, and then quickly washed with DEPC-treated water (0.1%). They were kept in 4% paraformaldehyde (PFA) solution (pH 7.3) and liquid nitrogen for histological and molecular studies respectively. They were then transported to the laboratory, and those for histological studies were maintained in 4% PFA solution (pH 7.3) for at least 7 d to preserve normal morphology. The tissue samples for molecular studies were stored in −80 °C and randomly assigned for end-point PCR, qRT-PCR, and Western blot studies. Each experiment was performed in triplicates. Table 1 shows all parameters for animal samples collected.

### 2.3. Histological Studies of Yellow-Cattle and Yak Testes and Determination of Testicular Index

Testes fixed in 4% PFA solution (pH 7.3) were processed using standard histological procedures. Briefly, testes were cut into blocks (0.5–1 mm^3^ each) and embedded in paraffin wax. Paraffin sections (4 μm) were dewaxed in dimethyl benzene and then processed with decreasing alcohol gradient. Samples were then treated in haematoxylin, hydrochloric acid, eosin, and increasing alcohol gradient, then finally in xylene. After processing, samples on glass slides were covered with mounting medium (neutral resin), then cover slides, and viewed under Olympus-DP73 optical microscope (Tokyo, Japan) for photographic images. Testicular morphology and structural differences among tissues were observed and compared.

The organ (testicular) index was determined as follows: Organ (testicular) index = organ weight/body weight × 100.

### 2.4. Primer Design, Amplification, and MTHFR CDS Sequencing

RT-PCR primers for y-c MTHFR and β-actin genes were designed based on conserved sequence homology from y-c (*Bos taurus*) available at the National Center for Biotechnology Information (NCBI) website for initial screening (reverse transcription PCR amplification reaction). Primers for cloning the initial fragment of MTHFR mRNA were designed according to the prediction of conserved sequences in other *Bos taurus* animals. Information on RT-PCR primers for y-c MTHFR and β-actin (as an internal control) are listed in Table 2.

### 2.5. Bioinformatics Studies

#### 2.5.1. Analysis and Alignment of DNA and Amino Acids Sequences

In this study, the y-c MTHFR sequence was analysed for nucleotide characteristics, protein translation, and sequence alignment with other mammalian species. Based on coding sequences available on the NCBI website (https://www.ncbi.nlm.nih.gov) analysis of y-c MTHFR nucleotide sequence was performed. Open reading frames (ORFs) encoding amino acids were detected in the DNA sequence. Nucleotide sequence of the DNA fragments was translated at https://web.expasy.org/translate, and the deduced amino acid sequence was analysed and compared with that of other mammals using NCBI BLASTP (https://www.ncbi.nlm.nih.gov) (Bethesda, MD, USA) and Expasy Protparam (https://web.expasy.org/protparam/) (Lausanne, Switzerland) programs. ClustalW (https://www.genome.jp/tools-bin/clustalw) and MEGA5.1 (https://www.megasoftware.net/citations) software was used to perform multiple sequence alignment (MSA) on eleven homologous sequences from different mammals.

#### 2.5.2. MTHFR Protein Secondary and 3D Structure Prediction

The SOPMA secondary structure prediction method (https://npsa-prabi.ibcp.fr/) was used to predict and construct the secondary structure of y-c MTHFR protein. Secondary structural elements based on hydrogen bonds within the molecule formed several recognizable “domains” of secondary structure like stems, hairpin loops, bulges, and internal loops. The base of the homology structure model, 3-dimensional (3D) structure of the protein was constructed using the Swiss-model software (https://swissmodel.expasy.org/). Similarities between y-c MTHFR and human MTHFR protein structures were also analysed. The bioinformatics program TMHMM (http://www.cbs.dtu.dk/services/TMHMM/) was also used to determine transmembrane helices in the protein and phosphorylation sites were detected using Expasy (https://www.expasy.org/proteomics) and NetPhos 3.1 (http://www.cbs.dtu.dk/services/NetPhos/). The molecular weight, isoelectric point (pI), charges, and hydrophobicity of MTHFR protein were determined using Expasy Protparam (https://web.expasy.org/protparam/) and CLCbio main workbench 20.0.4 software (Düsseldorf, Germany). The subcellular distribution of MTHFR proteins was determined using the PSORT II software (https://psort.hgc.jp/cgi-bin/runpsort.pl). The Heliquest analysis tool (www.heliquest.ipmc.cnrs.fr) was used to determine the polar amino acid composition of the first 18 amino acid. An amino acid scale was defined by a numerical value assigned to each type of amino acid. Hydrophilic/hydrophobic properties of MTHFR protein in y-c were also analysed [32]. The most widely used scales were hydrophobicity or hydrophilicity and secondary structure conformational parameter scales, however, many other scales based on different chemical and physical properties of amino acids are also available.

### 2.6. Extraction of Total RNA and Reverse Transcription

Total RNA from different testicular tissues were extracted by Trizol reagent according to the manufacturer’s instructions. Briefly, Trizol reagent (1.0 mL) was added to minced tissues (0.5 g) and incubated on ice for 5 min to allow nucleoprotein complexes to completely dissociate and subsequently generate RNA pellets. The RNA was reverse transcribed into cDNA and used for polymerase chain reaction (PCR). The cDNA synthesis reaction was achieved using RNA, 2 μL; Primer 2 oligo dt, 1 μL; Nuclease free-water, 2 μL; at 70 °C for 5 min, GoScript^TM^ 5x reaction buffer, 4 μL, MgCl_2_, 3 μL; Recombinant RNase in ribonuclease inhibitor, 0.5 μL; GoScript^TM^ reverse transcriptase, 1 μL; and Nuclease free-water, 5.5 μL. The complete mixture was run in a thermo cycler with the following conditions: 25 °C for 5 min, 42 °C for 1 h and 4 °C infinity, according to manufacturer’s instructions. The PCR products were electrophoresed on agarose gel (1.5%) using DNA Ladder DL2000. The synthesized cDNA was stored in −20 °C until it was used for further experiments.

### 2.7. qRT-PCR and MTHFR mRNA Expression Studies in Different Bovine Tissues

For relative quantitative gene expression, MTHFR mRNA expression levels in the testes of juvenile and adult y-c or yaks were compared. Total cDNA (200 ng) in a 20-μL reaction volume was analysed in LightCycler96 thermocycler using SYBR Green Premix DimerEraser and 100 nM of reverse and forward primers each. All RT-PCRs were run in triplicates with a non-template control to check for contaminations. The conditions of qRT-PCR were 3 min at 95 °C and 40 cycles each at 95 °C for 30 s, 60 °C for 30 s, and 72 °C for 15 s. Melting curve analysis was performed from 65 °C to 95 °C in 0.5 °C steps each lasting 5 s to confirm the presence of a single product and absence of primer-dimers. The relative expressions of target genes were calculated using 2^−ΔΔCt^ method with β-actin as the internal control.

### 2.8. Protein Extraction and Western Blot Analysis

Based on instructions from the literature, appropriate volumes of RIPA buffer (Solarbio, Beijing, China) together with PMSF were used to extract total protein from testicular tissues. After centrifugation, the supernatant containing total protein was collected and denatured with 4× SDS-PAGE loading buffer (Solarbio, Beijing, China). Denatured proteins were electrophoresed using SDS-PAGE (Bio-Rad, Irvine, CA, USA) and transferred to PVDF membranes (Millipore, Darmstadt, Germany). The membrane was blocked for 2 h at room temperature in PBST solution, containing 5% skim milk powder. It was then incubated with customized rabbit anti-MTHFR antibody (1:100, Bioss, Beijing, China), and mouse anti-β-actin antibody (1:2500; TransGen, Beijing, China) at 4 °C overnight. Corresponding secondary antibodies were used to incubate membranes for 1.5 h at room temperature after washing away primary antibody with PBST. Finally, ECL detection kit (Beyotime, Shanghai, China) was used for color reaction, and Amersham Imager 600 (GE Healthcare Life Sciences, Marlborough, MA, USA) was used to observe the resulting bands. β-actin was used as the control and the results were analyzed using Image J software v. 1.38 at https://imagej.nih.gov/ij/ (National Institutes of Health, Bethesda, MD, USA)

### 2.9. Statistical Analysis

All experiments were repeated at least three times, and data were presented as mean ± SD and analysed via independent samples *t*-test and one-way analysis of variance (ANOVA) from SPSS (Statistical Package for the Social Sciences 22.0) software (Chicago, IL, USA). *p* < 0.05 was considered statistically significant, and *p* = 0.01 considered extremely significant, statistically.

## 3. Results

### 3.1. Histological Evaluation of MTHFR Protein in Yellow-Cattle and Yak Testes and Determination of Testicular Index

**Figure 1:**
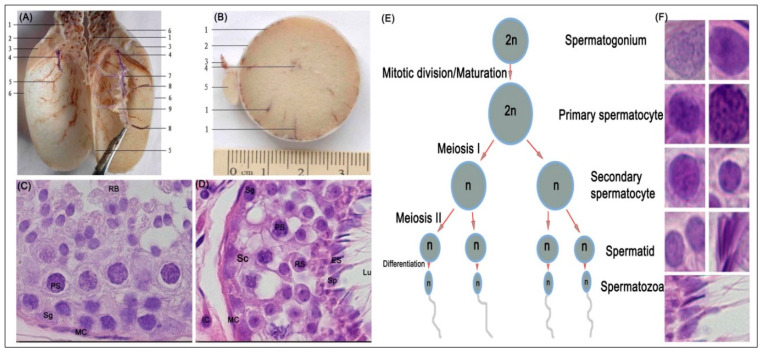
Morphologic and histologic study: (**A**) testis of yak longitudinally cut and without parenchyma testis; 1, pampiniform plexus; 2, head of epididymis; 3, epididymis; 4, efferent ductules; 5, vein; 6, tunica albuginea; 7, mediastinum testis; 8, arteries; 9, parenchyma testis. (**B**) Testis of yak (intersect), 1, blood vessel; 2, tunica albuginea; 3, ductus deferens; 4, mediastinum testis; 5, epididymis. (**C**,**D**) Representative haematoxylin & eosin (H&E) stained seminiferous epithelium section of yak testes (×1000), (**E**) stages in spermatogenesis; (**F**) representative structures of spermatogenesis in the yak testes. Lu, lumen; Sc, Sertoli cell; PS, primary spermatocyte; ES, Elongated spermatid, MC, myoid cells, RB, round body; Sg, Spermatogonia; RS, Round spermatid; Sp, Spermatozoa; IC, Interstitial cell.

Figure 1 shows a representative photomicrograph of yak testes and the various stages of spermatogenesis. Anatomical characteristics of testis in y-c (not shown) and yak were generally similar except for the weight or size, for which that of y-c was significantly higher or greater than yak (Figure 1A,B).

The body weight, testicular weight, and indices of y-c and yak are shown in Table 3. The seminiferous tubule epithelium was continuous and complete, and the germ cells had normal morphology and regular arrangement in all samples. Spermatogonia, primary spermatocytes, secondary spermatocytes, and spermatids were also observed in all samples (Figure 1C–F) and at various stages of spermatogenesis (E). However, in the adult testes, lumina in the seminiferous tubules were more developed and more spermatozoa were observed whereas in the juvenile bovines small lumina (not shown) were observed with the presence of few spermatozoa. In the adult y-c, the seminiferous tubules were loosely packed mostly with enlarged lumina. Through microscopy observations, we realized that the seminiferous tubules in juvenile y-c and yak testes occupied wider area of the testicular parenchyma than in adult y-c or yak which had larger lumina.

### 3.2. Characterization of the Full Coding Region of Yellow-Cattle MTHFR Gene

**Figure 2:**
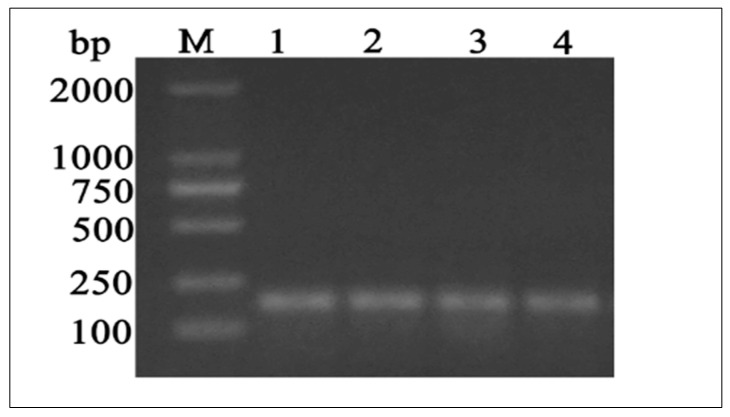
DNA gel electrophoresis of PCR products showing bands of MTHFR coding sequence in testes samples. M, marker, L1, L2, L3, and L4 stand for juvenile yak, juvenile yellow-cattle, adult yak, and adult yellow-cattle. bp: base pairs

Based on sequence homology, PCR primers were used to amplify a single 187 bp PCR fragment (Figure 2). In y-c, a sequence length of 2600 bp covered the full coding region. MTHFR gene sequence analysis revealed a 1968 bp ORF, with the 5′ and 3′—terminal UTR (untranslated regions) corresponding to 192 bp (initiation codon) and 2159 bp (termination codon) respectively. A comparison of y-c coding sequence with other organisms showed the following similarities: *Bos indicus*, 99.51%; *Bos mutus*, 98.68%; *Bubalus bubalis*, 98.47%; *Capra hircus*, 96.20%; *Ovis aries*, 96.08%; *Camelus bactrianus*, 88.05%; *Equus caballus*, 88.86%; *Canis lupus familiaris*, 87.94%; *Sus scrofa*, 89.11*%,* and *homo sapiens*, 87.35%. Y-c MTHFR showed the highest homology with wild yak (*Bos mutus*) and the lowest with horse (*Equus caballus*). ORF analysis of y-c (*Bos taurus*) MTHFR sequence is shown in Figure S1.

### 3.3. Molecular Composition of MTHFR Protein

**Figure 3:**
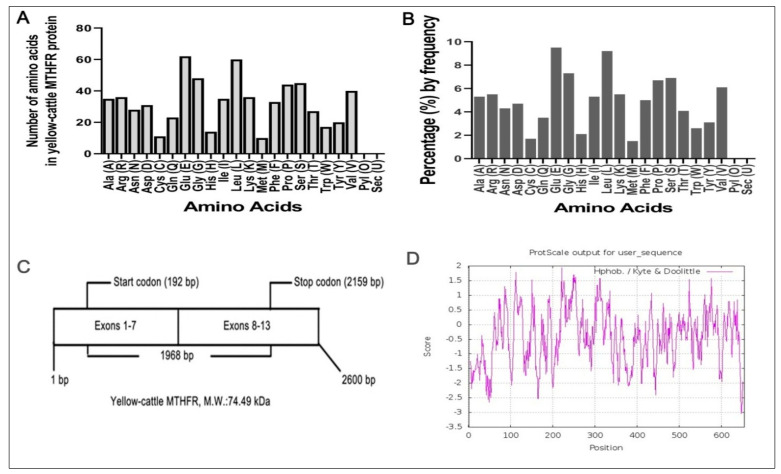
(**A**,**B**) Amino acid residues in yellow-cattle MTHFR protein, (**C**) Structure of MTHFR transcript, (**D**) Hydrophobicity analyses of the encoding product of yellow-cattle MTHFR protein.

Using Expasy Protparam software, we found that y-c MTHFR (molecular formula, C_3358_H_5143_N_895_O_985_S_21_) has 10,402 atoms with a molecular weight of 74.49 kDa. Amino acid number and percentage by frequency were determined (Figure 3A,B) and we found that MTHFR transcript had 13 exons encoding 655 amino acids residues (Figure 3C) with a theoretical isoelectric point (pI) of 5.30. Its N-terminal was composed of methionine (Met, M) with a half-life of 30 h in mammals, >20 h in yeast, and >10 h in *E. coli*. The total number of negatively (Asp + Glu) and positively (Arg + Lys) charged residues were 93 and 72, respectively. Also, the predicted protein contained 322 hydrophobic residues (Ala, Phe, Gly, Ile, Leu, Met, Pro, Val, and Trp, 49.16%), 154 hydrophilic residues (Cys, Asn, Gln, Ser, Thr, and Tyr, 23.51%) and 179 others (Asp, Glu, His, Lys, and Arg, 27.33%). MTHFR proteins’ instability index (II) was found to be 52.27 with an aliphatic index and grand average of hydropathicity (GRAVY) of 79.62 and −0.430, respectively. Figure 3D shows the hydrophobicity analyses of y-c MTHFR protein and comparison of y-c (*Bos taurus*) MTHFR protein with other MTHFR proteins from several, mostly similar mammals are shown in Table 4.

### 3.4. Multiple Sequence Alignment and Phylogenetic Analysis

**Figure 4:**
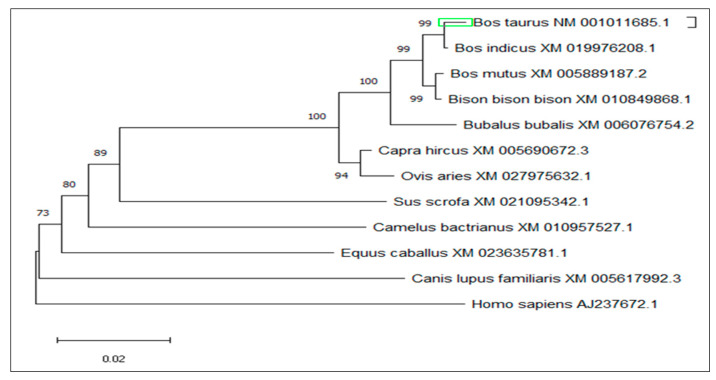
Phylogenetic tree of MTHFR among closely related species.

The amino acid sequence of y-c MTHFR was used as a template to identify homologous mammalian sequences using PSI-BLAST (https://www.ebi.ac.uk/Tools/sss/psiblast/) software. Eleven homologous sequences from different mammals were used for MSA through ClustalW and MEGA5.1 software. The output of MSA was color-coded according to nucleotide or amino acid identity and a phylogenetic tree (Figure 4) was constructed using amino acid sequences of MTHFR from all eleven mammalian species selected. MSA of amino acid sequences of closely related species are shown in Figure S2.

### 3.5. Analysis of the Physical and Chemical Characteristics of Yellow-Cattle MTHFR Protein

**Figure 5:**
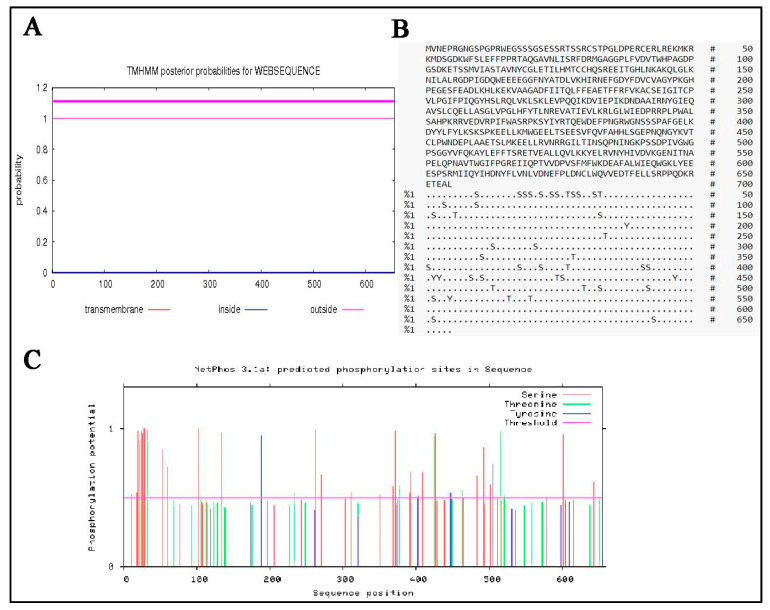
(**A**) Determination of transmembrane characteristics of yellow-cattle MTHFR, and (**B**,**C**) Phosphorylation sites in yellow-cattle MTHFR protein sequence.

The TMHMM software was used to predict transmembrane helices of MTHFR protein. It was found that y-c MTHFR had no transmembrane region (Figure 5A). The subcellular distribution of MTHFR proteins were as follows: cytoplasm (78.3%), nuclear (13.0%), mitochondrial (4.3%), and vesicles of the secretory system (4.3%). Also, phosphorylation sites (Figure 5B,C) in y-c MTHFR protein were predicted and analysed using NetPhos 3.1 software and we found that several phosphorylation sites existed in the protein. Phosphorylation scores greater than the threshold (0.500) potentially indicated the presents of phosphorylation sites. Predicted phosphorylation sites were marked with S, T, and Y for serine, threonine, and tyrosine, respectively (Figure 5B). Aspects of the chemical composition of MTHFR protein are shown in Figure S3.

### 3.6. Predicted Secondary and 3D Structure Characteristics of MTHFR Protein

**Figure 6:**
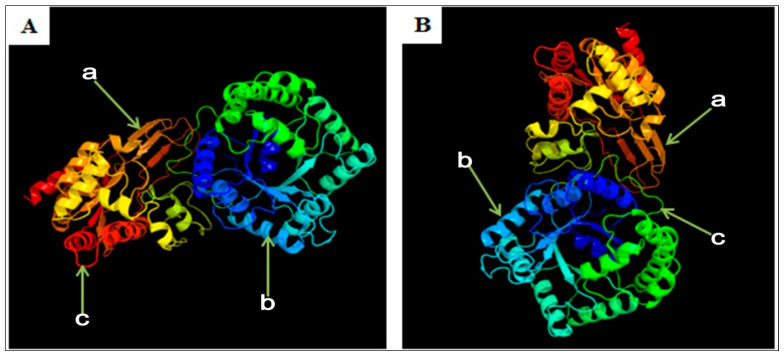
3D structure of MTHFR protein; (**A**) yellow-cattle MTHFR; (**B**) human MTHFR. a, extended strand; b, alpha-helix; c, random coil.

Using SOPMA software, the secondary structure of y-c MTHFR protein was predicted. Y-c MTHFR protein composed of alpha-helix 255 (Hh: 38.93%), extended strand 86 (Ee: 13.13%), beta-turn 37 (Tt: 5.65%), and random coil 277 (Cc: 42.29%). It was predicted to be more localized subcellularly in the cytoplasm. The 3D structure of y-c MTHFR was predicted with 91.61% sequence identity, modeled with 100.0% confidence and 90% coverage. The result showed general folding pattern and the 3D tertiary structure was highly similar to that of *Homo sapiens* MTHFR (with 97% sequence identity), which was modeled with 100.0% confidence and 87% coverage (Figure 6). Using Heliquest analysis tool, we found that polar amino acid composition of the first 18 amino acid sequence was 12, 4, and 4 for minimal number of polar residue + glycine, minimal number of uncharged residue (Ser and Asn), and minimal number of glycine respectively. Also, the maximum number of charged residues was 4 in both y-c and human MTHFR proteins. Interestingly, for every other 18 amino acid residue along the entire sequence, these parameters changed, and the change was consistent with human MTHFR protein suggesting a high level of similarity in character and function. Predicted secondary structure of *Bos taurus* MTHFR are shown in Figure S4.

### 3.7. Expression of MTHFR mRNA and Protein in Yellow-Cattle and Yak Testes

**Figure 7:**
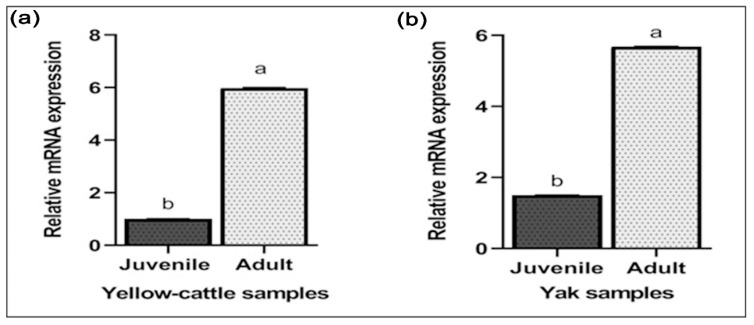
RT-qPCR results of MTHFR mRNA expression in testes of juvenile and adult yellow-cattle (y-c) or yak. (**a**) MTHFR relative mRNA expression in juvenile and adult yellow-cattle testes. (**b**) MTHFR relative mRNA expression in juvenile and adult yak testes. The mRNA levels were normalized using β-actin mRNA as an internal control. (y-c, *n* = 3, yak, *n* = 3) for ≤2 years juvenile and (y-c, *n* = 3, yak, *n* = 4) for ≥3 years adult y-c or yak. The significant differences among mRNA levels were analyzed by SPSS one way ANOVA and samples *t*-test while statistical significance was defined as *, *p* < 0.05; **, *p* < 0.01. Different letters indicate significant difference (*p* < 0.05, *p* < 0.01).

**Figure 8:**
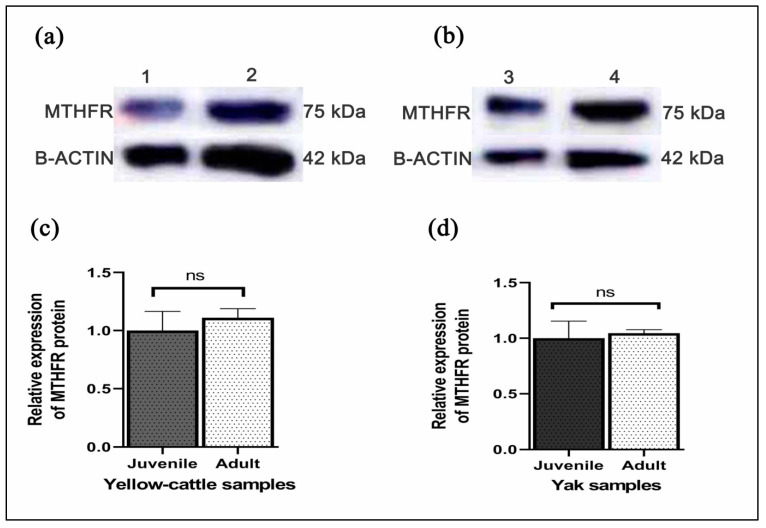
(**a**,**b**) Representative Western blot bands of MTHFR and β-Actin proteins in juvenile and adult yellow-cattle (y-c) or yak testes; juvenile (1: y-c; 3: yak), adult (2: y-c; 4: yak). (**c**) MTHFR relative mRNA expression in juvenile and adult yellow-cattle testes. (**d**) MTHFR relative mRNA expression in juvenile and adult yak testes. (y-c, *n* = 3, yak, *n* = 3) for ≤2 years juvenile and (y-c, *n* = 3, yak, *n* = 4) for ≥3 years adult y-c or yak. The significant differences among protein levels were analyzed by SPSS one way ANOVA and samples *t*-test while statistical significance was defined as *, *p* < 0.05. ns indicates no significant difference (*p >* 0.05).

Y-c MTHFR mRNA in testicular tissues of y-c and yaks were investigated by real-time polymerase chain reaction (RT-PCR). As shown in Figure 7a,b relative mRNA expression levels in both adult y-c and yak testes were highly significant compared to their juvenile counterparts and this expression level was 5.586 folds higher in the adult y-c testes than the juvenile ones (1.000) (*p* < 0.05, *p* < 0.01). In a like-wise manner, the expression level in the adult yak testes was 5.686 folds higher than that in the juvenile ones (1.500) (*p* < 0.05, *p* < 0.01). This shows that the highest expression level of MTHFR mRNA was observable during adulthood in both species. On the other hand, MTHFR protein expression was higher but not statistically significant in adult yellow-cattle and yak testes compared to their juvenile ones. MTHFR protein expression tendencies were consistent at the different ages between y-c and yak. According to our data, levels of MTHFR were high in almost all adult testes studied compared to the juvenile ones (Figure 8a–d).

## 4. Discussion

For the first time, this study evaluated testicular histopathology, and analysed DNA sequence, amino acids, relative mRNA expression, and predicted protein characteristics of MTHFR in the testes of juvenile and adult y-c or yak. It highlighted the unique characteristics of this enzyme in bovine testes and examined roles MTHFR could play in the physiology of bovine spermatogenesis. MTHFR is pivotal in folate metabolism and controls the relative distribution of one-carbon moieties between two important biochemical pathways that support general housekeeping methylation reactions and purine/pyrimidine biosynthesis. Precise control of MTHFR expression may therefore be essential to ensure that the optimum balance between the above pathways is tailored to tissue-specific requirements. Characterization of the structure of its mRNA in both human and mouse has also provided a foundation for studies on the regulation of MTHFR [33].

In bovines, testicular growth increases markedly between 7 and 10 months of age [34]. Anatomical characteristics of testis in y-c and yak were generally similar except for the weight or size for which that of y-c was significantly higher or greater than yak. Analysis of testicular structure revealed that healthy juvenile and adult y-c or yak testes had a continuous arrangement of seminiferous tubule epithelium and germ cells also had normal morphology and regular arrangement. Primary spermatocytes and early spermatids were also observed in all tissues. The lumina (with spermatocytes) in the seminiferous tubules were more developed in the adult testes than in the juvenile ones. This could probably be due to age-related physiological changes. In bovines, lumen appears around the age of 20 weeks and increase from 2% to 98 µm by the age of 32 weeks [35].

Bioinformatics analysis showed that y-c MTHFR nucleotide sequence had several ORFs including a 1968 bp, with 5′ and 3′—terminal UTR (untranslated regions) corresponding to the 192 bp (initiation codon) and 2159 bp (termination codon) respectively. This was different in the case of human MTHFR as reported by Homberger et al. [36]. In this study, we found that y-c MTHFR had 10,402 atoms with a molecular weight of 74.49 kDa whereas in human studies, 70 to 77 kDa was reported. Six hundred and fifty-five (655) amino acids constituted MTHFR protein, and a sequence length of 2600 bp covered the full coding region with 13 exons whereas in humans, MTHFR had sequence length of 2196 bp with 11 exons encoding 679 amino acids in one instance. The human MTHFR encoded three putative polypeptides of 657, 679, and 698 amino acids [36]. Y-c MTHFR coding sequence was highly similar with that of other organisms and of all species considered, the highest level of homology was observed in wild yak (*Bos mutus*) and the lowest in horse (*Equus caballus*); this suggests a common evolutionary origin.

The MTHFR protein N-terminal was composed of methionine (Met, M) with a half-life of 30 h in mammals. It is reported that more than 80% of proteins of any given proteome has methionine at its N-terminal [37]. Protein N-terminal methionine (NM) is an essential cotranslational factor that occurs in the cytoplasm of all organisms and in two organelles (i.e., mitochondria and plastids) capable of protein synthesis [38,39]. Amino acids have their unique and specific chemical characteristics enabling them to perform exclusive roles in protein structure and function. Depending on the inclination and proximity of the side chains to water, amino acids can be classified as hydrophobic (lowly inclined to be in contact with water), polar and charged (energetically favoured to be in contact with water) [40]. In our study, we found that the predicted MTHFR protein contained 322 hydrophobic and 154 hydrophilic residues and the total number of negatively (Asp + Glu) and positively (Arg + Lys) charged residues were 93 and 72 respectively. Instability index (II) was found to be 52.27 with an aliphatic index and grand average of hydropathicity (GRAVY) of 79.62 and −0.430 respectively. These data suggest that y-c MTHFR gene codes for an unstable and very reactive protein.

In this study, it was also found that y-c MTHFR had no transmembrane region, thus belonging to the extra-membrane category of proteins. Proteins within a cell are often localized to specific cellular compartments, such as the cytoplasm, nucleus, mitochondria, plasma membrane, or vesicles, and their specific localization can provide crucial information about the function of the protein [41]. The distribution of MTHFR proteins was subcellularly predicted to be more in the cytoplasm (78.3%). Subcellular localization determines the access of proteins to interacting partners and the post-translational modification machinery, which enables the integration of proteins into functional biological network [42]. It has also been demonstrated that the abnormalities of subcellular locations of protein are potentially involved in the pathogenesis of many human diseases [42,43,44,45].

Protein phosphorylation is the most abundant form of cellular regulation, essentially organizing all cell functions, including metabolism, proliferation, differentiation, motility, survival, and death. Protein phosphorylation is a post-translational modification of proteins whereby a phosphate group is covalently attached to either a serine, threonine or tyrosine residue [46,47]. Phosphorylation sites in y-c MTHFR predicted protein were analysed and we found that several phosphorylation sites existed in the protein. Scores greater than the threshold (0.500) indicated potential presents of phosphorylation sites, however, scores just above the threshold implied that the confidence for categorizing the site as phosphorylated was low.

To a large extent, secondary (α-helices and β-sheet) and tertiary structures of proteins are defined by amino acids, although the effect of local environment on structure stabilization is also crucial. The α-helix is considered to be the most abundant form of secondary structure, accounting for about 31% of amino acid secondary structure states [48]. In our study, we found that the secondary structure of y-c MTHFR protein was made of α-helix 255 (Hh: 38.93%), extended strand 86 (Ee: 13.13%), beta-turn 37 (Tt: 5.65%), and random coil 277 (Cc: 42.29%). The predicted 3D structure of y-c MTHFR protein had 91.61% sequence identity, modeled with 100.0% confidence and 90% coverage. The result showed general folding pattern and the 3D structure was highly similar to that of *Homo sapiens* MTHFR.

A comprehensive understanding of the cell biology, protein structure, and genetics of spermatogenesis in most species is arduous because it occurs within a complex testicular environment characterized by the intimate association of developing sperms with accessory cells [49]. Nevertheless, in recent years, advances in genomics have greatly improved our knowledge of spermatogenesis by identifying numerous genes/proteins essential for the development of functional male gametes. Large-scale analyses of testicular function have deepened insights into normal and pathological spermatogenesis [50].

Studying MTHFR mRNA expression in testicular tissues of juvenile and adult y-c or yak, we found a relatively high expression level in the adult testes compared to the juvenile ones. This suggests that with the passage of time or maturity, there are increased/pronounced activities of MTHFR enzyme in the testes to promote its core functions, which is the production of spermatozoa through spermatogenesis. This could partly explain why the highest MTHFR mRNA expression levels were observed during adulthood in healthy bovines. Liu et al. [51], working on bovines, reported that mRNA and protein expression were mostly consistent at different developmental stages. They added that mRNA expression levels were significantly higher for some testicular genes in almost all developmental stages of y-c and yak [51]. Chen et al. in 2001 also reported that animal model studies suggested that MTHFR plays a critical role in spermatogenesis due to exceptionally higher activity in adult mouse testes [26]. Meanwhile, Kelly et al. [20] observed that inactivation of MTHFR results in infertility with abnormal testicular histology characterized by absence of germinal cells and spermatogenesis arrest in male mice [20]. At the transcript level, the mRNA expression of MTHFR was significantly higher in adult y-c and yaks compared to their juvenile ones. Relating the expression characteristics to spermatogenesis, we speculate that in the testes of healthy matured bovines, the activities of MTHFR enzymes are high and this had a direct correlation with onset and maintenance of spermatogenesis and the production of spermatozoa. As part of ongoing efforts to study MTHFR deficiency in homocystinuria and in multifactorial diseases, in recent times, the isolation of human MTHFR genes and analysis of the gene structure are being carried out. Daubner and Matthews in 1982 reported that MTHFR polypeptide of 70 kDa was observed in some human tissues on Western blots, and a larger isozyme (77 kDa), corresponding to an estimated size of porcine polypeptide, was also observed in all examined tissues [27,52]. In our study, MTHFR protein expression was higher but not statistically significant in adult yellow-cattle and yak compared to their juvenile ones. MTHFR protein expression tendencies were consistent at different ages between y-c and yak. Proteins of this enzyme are more localized subcellularly in the cytoplasm of cells. According to our data, levels of MTHFR were higher in almost all adult testes studied compared to the juvenile ones. These expression patterns in the testes could be due to age differences and physiological changes, thus establishing a relationship with spermatogenesis.

We have studied the DNA sequence, amino acids, protein structure, and the mRNA expression characteristics of MTHFR in healthy juvenile and adult y-c or yak testes. Elsewhere, it was reported that under normal regulation, MTHFR contributes to testes development, spermatogenesis, and male fertility. We therefore speculate that since male cattle-yak, an F1 hybrid between y-c and yak is sterile in the face of several desired heterosis, there could be mutations/polymorphism/epigenetic changes in the DNA or amino acid sequences in cattle-yak testes leading to impairment of normal protein function. These could contribute to cattle-yak sterility. Our next research project, therefore, will focus on unraveling the bioinformatics and expression characteristics of MTHFR in the testes of cattle-yak and compare with that of y-c and yaks.

## 5. Conclusions

In conclusion, although this study needs to be explored further, we have conducted a more systematic and in-depth study on the histologic structure, bioinformatics profile, MTHFR relative mRNA and protein expression in the testes of juvenile and adult y-c or yaks. It gives a clearer and vivid understanding of the testicular structure, DNA nucleotide and amino acids sequence, MTHFR mRNA and protein expression characteristics in y-c or yak. We have shown that anatomically, the structure of y-c and yak testes are similar except the weight/size for which y-c testis was higher/greater than that of yak. Also, the secondary and 3D protein structure of MTHER in y-c was similar to that of humans but differed in the number of nucleotides, amino acids, and some physico-chemical characteristics. More so, MTHFR expressed in both juvenile and adult y-c and yak testis, however, MTHFR mRNA and protein expressions were higher in adult y-c or yak testes compared to that of the juvenile ones. These results will not only provide an understanding of the testicular structure of y-c and yaks but also provide the basis for investigations into the regulatory function of MTHFR in testes physiology and additionally enhance knowledge on investigations into testicular development and spermatogenesis in bovine species. It will also set the stage for studies on MTHFR gene in relation to hybrid male sterility in cattle-yak.

## Figures and Tables

**Table 1:** Parameters for animal samples collected.

Animal Species	Number of Animals (Testes)	Age Group	Age (Year)	Source (All in China)	Application
Yellow-cattle	3 (6)	Juvenile	1–2	Wuwei city	Histology and molecular biology studies
	3 (6)	Adult	3		
Yak	3 (6)	Juvenile	1	Linxia city	
	4 (8)	Adult	4		

**Table 2:** Primer sequences of target and house-keeping genes.

Primer Name	Sequence (5′ to 3′)	T*m* (°C)	Product Size (bp)	Accession No
MTHFR F-1	TCGTGGATGTGAAGGGTGAA	55.40	187	NM_001011685.1
MTHFR R-1	TCCTCCTCATACAGCTTGCC	57.45		
MTHFR F-2	GGAGAGAGCTTTGAGGCTGA	57.45	185	NM_001011685.1
MTHFR R-2	AGGGAGTGGTAGCCCTGAAT	57.45		
β-Actin F	GCAATGAGCGGTTCC	56.00	141	XM_005887322.2
β-Actin R	CCGTGTTGGCGTAGAG	56.00		

Tm: Melting temperature, bp: base pairs.

**Table 3:** Age, body weight, testicular weight, and index of juvenile and adult y-c and yak.

Animal Specie	Age Group	Age (Years)	Body Weight (kg) at Slaughter	Testicular Weight (g)	Testicular Index (g/kg × 100)
Yellow-cattle	Adult	3	569.67 ± 9.00	310.53 ± 5.85	55.01 ± 0.01 ^a^
	Juvenile	1–2	275.47 ± 7.64	117.21 ± 8.17	42.52 ± 0.02 ^b^
Yak	Adult	4	396.19 ± 9.43	152.28 ± 8.43	38.42 ± 0.01 ^c^
	Juvenile	1	292.57 ± 5.59	102.59 ± 4.41	35.06 ± 0.01 ^d^

Values are expressed as means ± SD; (y-c, *n* = 3, yak, *n* = 3) for ≤2 years juvenile and (y-c, *n* = 3, yak, *n* = 4) for ≥3 years adult y-c or yak. Values not sharing common superscript differ significantly at *p* < 0.05.

**Table 4:** Comparison of yellow-cattle (*Bos taurus*) MTHFR and other MTHFR proteins from various, mostly similar, mammals (with high percentage identity value of between 100 to 89.02%).

Animal Species MTHFR	Accession Number	No. of Amino Acid Residue	Total Score (%)	Identity (%)	Query Cover (%)	E-Value	pI	Molecular Weight
*Bos taurus*	NP_001011685.1	655	1357	100.00	100	0.0	5.30	74.49
*Bos mutus*	XP_005889249.1	696	1349	99.24	100	0.0	5.44	78.80
*Bos indicus*	XP_019831767.1	696	1347	99.08	100	0.0	5.44	78.78
*Bison bison*	XP_010848171.1	695	1348	99.24	100	0.0	5.44	78.72
*Capra hircus*	XP_005690729.2	799	1342	98.63	100	0.0	5.78	89.66
*Ovis aries*	XP_027831433.1	655	1342	98.63	100	0.0	5.18	74.52
*Bubalus bubalis*	XP_006076816.2	655	1341	98.47	100	0.0	5.22	74.58
*Camelus bactrianus*	XP_010955831.1	655	1254	93.58	99	0.0	5.23	74.67
*Canis lupus familiaris*	XP_005618050.1	656	1215	91.02	100	0.0	5.22	74.45
*Equus caballus*	XP_005607580.1	655	1231	92.20	99	0.0	5.11	74.45
*Sus scrofa,*	XP_020951001.1	700	1248	89.02	99	0.0	5.39	78.94
*Homo sapiens*	CAB41971.1	679	1209	89.92	99	0.0	5.30	76.88

E-value: Expect value, pI: isoelectric point.

## Supplementary Materials

The following are available online at https://www.mdpi.com/2076-2615/10/10/1731/s1, Figure S1. ORF analysis of yellow-cattle (*Bos taurus*) MTHFR sequence, Figure S2. Multiple sequence alignment of amino acid sequences of closely related species, Figure S3. Aspects of the chemical composition of yellow-cattle MTHFR protein, Figure S4. Predicted secondary structure of *Bos taurus* MTHFR.
