# Supplementary material for: Histological Analysis, Bioinformatics Profile, and Expression of Methylenetetrahydrofolate Reductase (MTHFR) in Bovine Testes
Source: Animals (Basel). 2020 Sep 23;10(10):1731. doi: 10.3390/ani10101731 (PMC7598625; doi:10.3390/ani10101731)
Supplement: Supplementary file 1 [file animals-10-01731-s001.pdf]

Supplementary figures

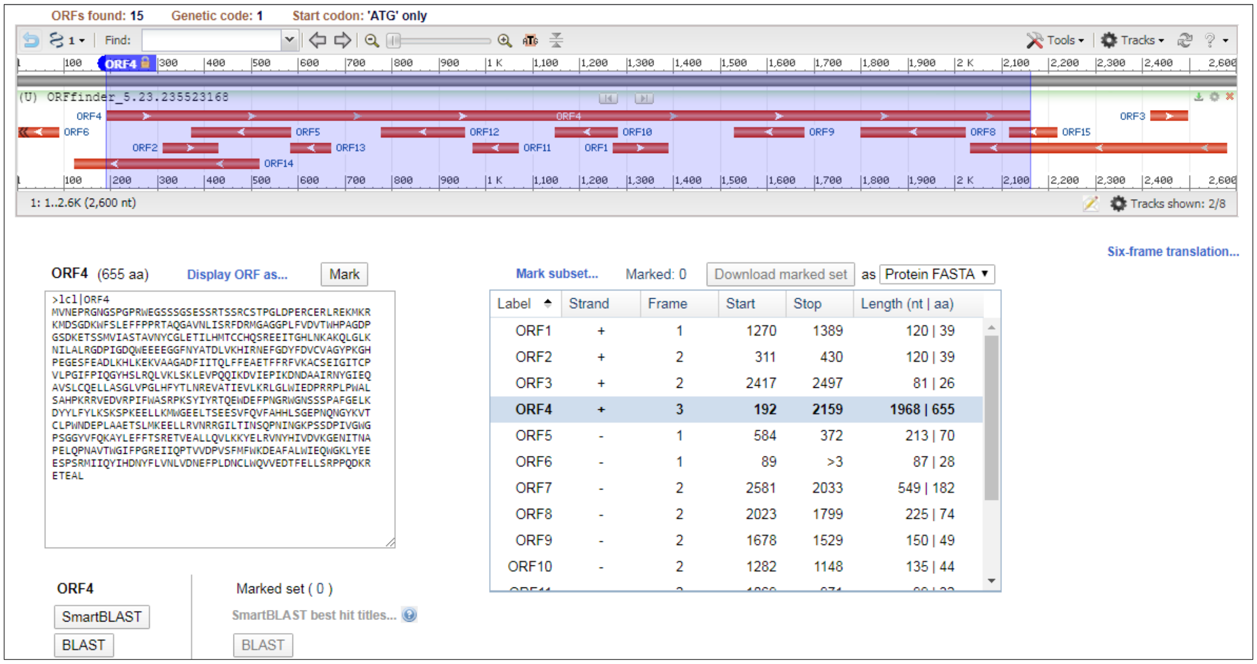

Figure S1. ORF analysis of yellow-cattle (*Bos taurus*) MTHFR sequence.

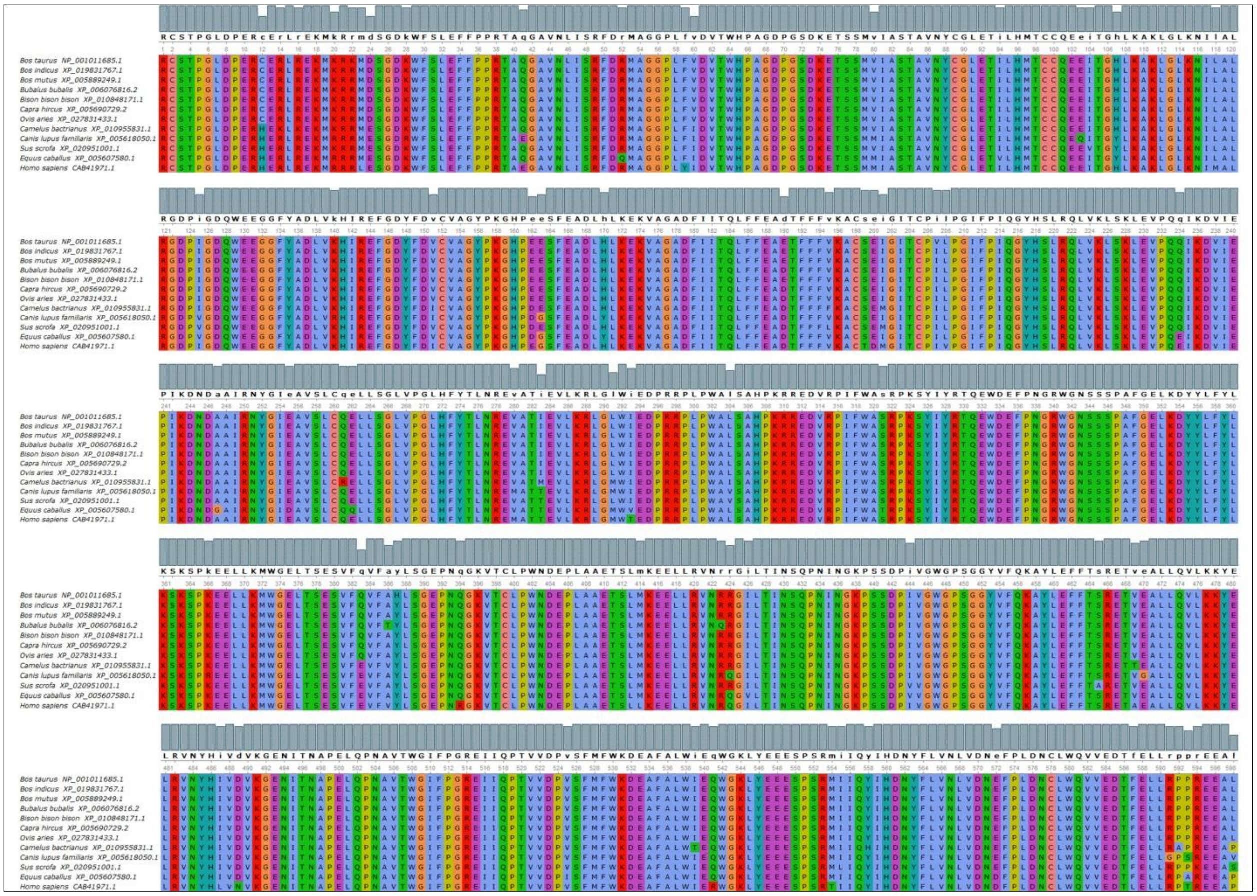

Figure S2. Multiple sequence alignment of amino acid sequences of closely related species.

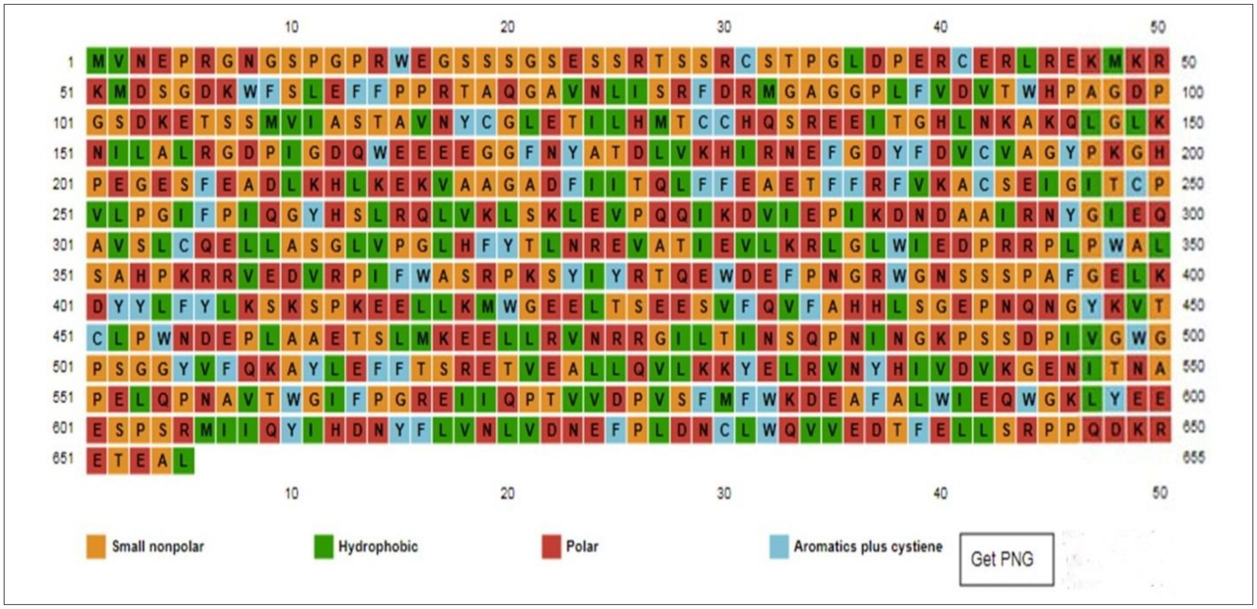

Figure S3. Aspects of the chemical composition of yellow-cattle MTHFR protein.

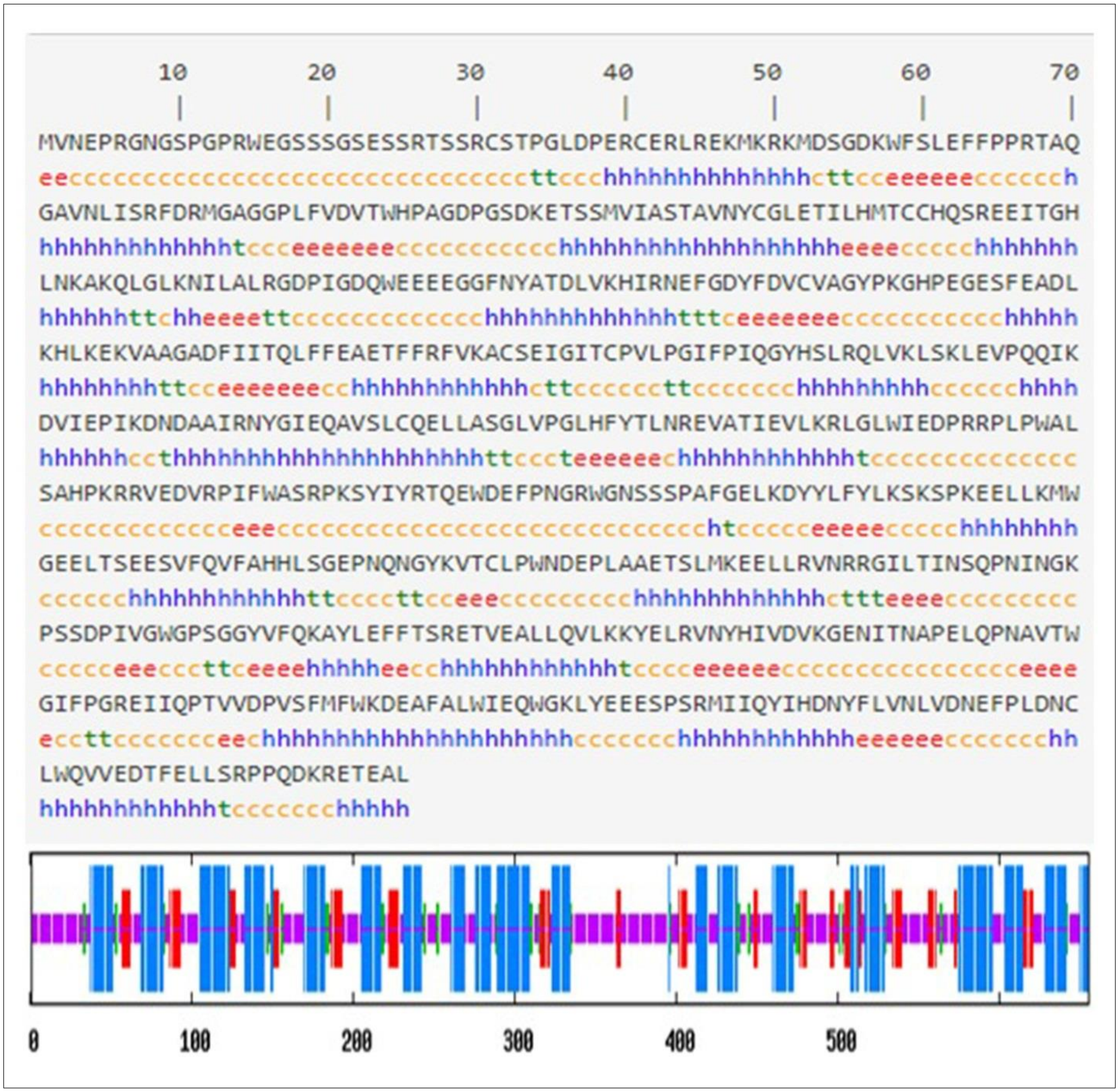

Figure S4. Predicted secondary structure of *Bos taurus* MTHFR. The long vertical zone: Alpha helix (h); The middle vertical zone: Extended strand (e); The short vertical zone: Random coil (c). The secondary structure was predicted by SOPMA online software.
